# Trilateral overlap of tuberculosis, diabetes and HIV-1 in a high-burden African setting: implications for TB control

**DOI:** 10.1183/13993003.00004-2017

**Published:** 2017-07-20

**Authors:** Tolu Oni, Natacha Berkowitz, Mmamapudi Kubjane, Rene Goliath, Naomi S. Levitt, Robert J. Wilkinson

**Affiliations:** 1 Division of Public Health Medicine, School of Public Health and Family Medicine, University of Cape Town, Cape Town, South Africa; 2 Wellcome Centre for Infectious Disease Research in Africa, Institute of Infectious Disease and Molecular Medicine, University of Cape Town, Cape Town, South Africa; 3 Division of Diabetes and Endocrinology, Dept of Medicine, Groote Schuur Hospital and Chronic Disease Initiative for Africa, Cape Town, South Africa; 4 The Francis Crick Institute, London, UK; 5 Dept of Medicine, Imperial College London, London, UK; 6 Joint senior authors

## Abstract

The diabetes mellitus burden is growing in countries where tuberculosis (TB) and HIV-1 remain major challenges, threatening TB control efforts. This study determined the association between TB and diabetes/impaired glucose regulation in the context of HIV-1.

A cross-sectional study was conducted at a TB clinic in Cape Town (South Africa). Participants were screened for diabetes and impaired glucose regulation using fasting plasma glucose, oral glucose tolerance test and glycated haemoglobin (HbA1c).

414 TB and 438 non-TB participants were enrolled. In multivariable analysis, diabetes was associated with TB (OR 2.4, 95% CI 1.3–4.3; p=0.005), with 14% population-attributable risk fraction; however, this association varied by diagnostic test (driven by HbA1c). The association remained significant in HIV-1-infected individuals (OR 2.4, 95% CI 1.1–5.2; p=0.030). A high prevalence of impaired glucose regulation (65.2% among TB cases) and a significant association with TB (OR 2.3, 95% CI 1.6–3.3; p<0.001) was also found.

Diabetes and impaired glucose regulation prevalence was high and associated with TB, particularly in HIV-1-infected individuals, highlighting the importance of diabetes screening. The variation in findings by diagnostic test highlights the need for better glycaemia markers to inform screening in the context of TB and HIV-1.

## Abstract 2

**DM/TB association is significant in HIV infection, but more accurate glycaemia markers at TB diagnosis are needed** http://ow.ly/ZXoS30aPz22

## Introduction

Tuberculosis (TB) remains a health challenge in most low- and middle-income countries, including those in sub-Saharan Africa. Poverty, smoking, alcoholism, HIV-1 and diabetes mellitus are known drivers of the TB epidemic [1]. Diabetes triples the risk of TB and is associated with adverse outcomes [2–4]. The TB/diabetes association is increasingly important in low- and middle-income countries experiencing a growing burden of noncommunicable disease. According to the 2013 Global Burden of Disease Study, HIV-1, TB and diabetes rank first, third and ninth among the top 10 causes of life-years lost in South Africa [5].

The prevalence of diabetes among TB patients varies by geographic region and background diabetes population in the general population, with a higher prevalence reported in South Asian (25.3–44%) and Latin American (14–39%) populations [6–12]. In sub-Saharan Africa, diabetes prevalence in TB patients ranges from 8.5% to 16.4%, although a large proportion of diabetes in sub-Saharan Africa remains undiagnosed [13–17]. Of note, most studies have used fasting or random blood glucose to diagnose diabetes, which may underestimate the true prevalence. A study in South Africa estimated the general population prevalence of diabetes and impaired glucose tolerance to be 12.3% and 11.2%, respectively [18].

The increased risk of TB associated with diabetes is well documented. However, this risk is variable dependent on the population studied. A systematic review that reported a 3-fold increased risk of TB in diabetes patients did not include studies from sub-Saharan Africa where there are competing TB risk factors such as HIV-1 [2]. Recent studies in African settings showed increased odds of TB among diabetes patients with the exception of one study [19], possibly due to the low background prevalence of diabetes. A Ugandan study found HIV-1-infected TB patients to have reduced odds of diabetes compared with HIV-1-uninfected patients [20] and a Tanzanian study showed a stronger TB/diabetes association observed among HIV-1-uninfected (OR 4.23, 95% CI 1.54–11.57) compared with HIV-1-infected (OR 0.14, 95% CI 0.01–1.81) individuals [21]. The reasons for this apparent incongruity in the associations between TB, diabetes and HIV-1 are unclear as there are only a limited number of published studies [22].

The only South African study that assessed the prevalence of diabetes, using the oral glucose tolerance test (OGTT), among TB patients was conducted in 1980, when South Africa was 42% urbanised (compared with 62% urbanised today), and reported a 2.1% prevalence [16]. South Africa has the largest antiretroviral therapy (ART) programme globally and is ranked as having the sixth highest TB incidence globally [23, 24]. Given this HIV-1/TB burden, reducing the TB burden in South Africa is a priority in the global fight against TB. The strong association between HIV-1 and TB, dysglycaemic effects of some ART drugs, especially protease inhibitors and nonnucleoside reverse transcriptase inhibitors [25, 26], and the emerging diabetes epidemic highlight the importance of investigating the association between TB, diabetes and HIV-1 in this setting, the results of which will have implications for TB control strategies. This study investigated the prevalence of diabetes and impaired glucose regulation (IGR), the association between TB and diabetes/IGR, and the population-attributable risk of TB due to diabetes in South Africa.

## Methods

### Study setting and population

In South Africa, TB patients in the public sector are largely screened and treated in TB clinics. This study was conducted at the largest TB clinic in Khayelitsha, a peri-urban township of around 390 000 predominantly black Africans, in Cape Town, Western Cape province. In this province, diabetes, HIV-1 and TB rank as the first, third and fourth leading causes of death, respectively [27]. The 2012 HIV-1 antenatal prevalence in Khayelitsha was 34% (95% CI 31.0–36.6%) (Western Cape Dept of Health, Cape Town, South Africa; 2012 Antenatal Survey, unpublished data) and the 2015 TB case notification rate was 917 per 100 000 population (V. de Azevedo, City Health Manager, Khayelitsha, Cape Town, South Africa; personal communication), with a 60% HIV-1/TB co-infection rate [28].

### Study design and sampling

We conducted a cross-sectional study on consecutive patients with respiratory symptoms presenting to the clinic from July 2013 from August 2015. Patients were eligible if they provided consent, were aged ≥18 years and had not received >48 h of TB chemotherapy. Those who were critically ill and in need of emergency clinical care were ineligible as they were too physically unwell to give informed consent. The study was approved by the University of Cape Town Human Research Ethics Committee (HREC 403/2011). Assuming a 3-fold higher diabetes prevalence in HIV-1-uninfected TB cases, 7% diabetes prevalence in HIV-1-uninfected individuals, 70% of TB cases HIV-1-co-infected and 80% power, the study aimed to recruit 400 TB and 400 non-TB participants.

### Study procedures

#### Case definitions

TB cases were diagnosed according to South Africa guidelines with the GeneXpert system (Cepheid, Sunnyvale, CA, USA) and analysed in a centralised national health laboratory [29]. Non-TB participants were those with a negative TB diagnosis and resolution of respiratory symptoms. All participants were encouraged to undertake an HIV-1 test and were tested for diabetes using all three of the following: fasting plasma glucose (FPG), a 2-h OGTT and glycated haemoglobin (HbA1c). Diabetes diagnosis was defined as self-reported diabetes, FPG ≥7.0 mmol·L^−1^, OGTT ≥11.1 mmol·L^−1^ or HbA1c ≥6.5% [30, 31]. IGR was defined as FPG 5.5–<7.0 mmol·L^−1^, OGTT 7.7–<11.1 mmol·L^−1^ or HbA1c 5.7–<6.5%.

#### Measurements

Venous blood was drawn from the antecubital vein at 0 (after an overnight fast) and 120 min in evacuated fluoride (glucose) and EDTA (HbA1c) tubes. All blood samples were processed on the day of collection at a centralised national health laboratory using standardised operating procedures of the cobas c311 (Roche/Hitachi, Basel, Switzerland) system analyser assay. Weight, height and waist circumference were measured using standardised techniques [32]. Body mass index (BMI) was categorised as underweight <18.5 kg·m^−2^, normal 18.5–24.9 kg·m^−2^, overweight 25–29.9 kg·m^−2^ and obese ≥30 kg·m^−2^ [32]. The cut-point for high waist circumference was ≥94 cm for males and ≥88 cm for females [32]. Hypertension was defined as a single measured blood pressure of systolic blood pressure >140 mmHg or diastolic blood pressure >90 mmHg [31], or a pre-existing diagnosis.

#### Questionnaire

Chronic disease risk factors were ascertained using the STEPs instrument [33]. Information on socioeconomic, demographic and medical history was collected using a researcher-administered questionnaire.

### Statistical analysis

Medians (interquartile ranges (IQRs)) and proportions were used to summarise continuous and categorical variables, respectively. Chi-squared and Fisher's exact tests assessed associations between categorical variables. The Mann–Whitney test was used to compare medians between two groups and the Kruskal–Wallis test for more than two groups. A multivariable logistic regression model for the association between TB and diabetes was built manually, using forward selection, controlling for potential confounding variables. In order to retain statistical power and reduce potential biases, in the regression analysis involving the key variable HIV serostatus, multiple imputation was used to impute HIV serostatus for 50 participants with unknown HIV-1 status. Imputation using the chained equations method was implemented in Stata (StataCorp, College Station, TX, USA) using the “ice” command. Five imputed datasets were created. The “mi” Stata command was used to perform logistic regression analysis on the combined imputed datasets. The base model included *a priori* confounding variables of age and sex. Variables associated with TB (p<0.10) on univariable analysis were then added, sequentially including variables that improved the model based on the significant lowering of the Akaike Information Criterion in nonnested model comparisons. Potential effect modification between variables was determined by exploring the statistical significance of interaction variables. The fit of the model was assessed using Pearson's goodness-of-fit test with p>0.05 indicating a good fit. The population-attributable risk was calculated as: AR_pop_diabetes=p(diabetes)(OR–1)/(1+p(diabetes)(OR–1)), where p(diabetes) is general population diabetes prevalence and OR is the adjusted odds ratio [34]. Statistical significance was set at p<0.05. All data were analysed using Stata version 13.0.

## Results

### Baseline characteristics

986 participants were recruited. 48 participants (4.9%) were excluded as they had an undetermined TB status (TB could not be confirmed or excluded). A further 86 participants did not complete diabetes screening at baseline (did not return for fasting bloods). 852 participants were therefore included in the final analyses: 414 TB cases and 438 non-TB participants. The overall median (IQR) age of participants was 38 (31–47) years, with 53% male. The overall prevalence of HIV-1 was 61.2% (95% CI 29.9–36.2%), and was significantly higher in participants with TB (*versus* non-TB) and in females (table 1). Compared with those without TB, TB cases were younger, with a lower prevalence of obesity and a greater proportion of men. Of the 414 TB cases, nine had rifampicin resistance (2.2%), all of whom did not have diabetes. Table 1 summarises baseline characteristics of participants, stratified by TB status.

**TABLE 1:** Baseline sociodemographic, anthropometric and comorbidity characteristics by tuberculosis (TB) status

	**Non-TB**	**TB**	**Total^#^**	**p-value**
**Subjects**	438	414	852	
**Age group years**				<0.001*
18–24	16 (3.7)	43 (10.4)	59 (6.9)	
25–34	126 (28.8)	151 (36.5)	277 (32.5)	
35–44	126 (28.8)	138 (33.3)	264 (31.0)	
45–54	95 (21.7)	53 (12.8)	148 (17.4)	
≥55	75 (17.1)	29 (7.0)	104 (12.2)	
**Age years**	41 (32–50)	36 (30–43)	38 (31–47)	<0.001*
	(range 20–80)	(range 18–80)	(range 18–80)	
**Female n=848**	224 (51.3)	175 (42.6)	399 (47.0)	0.011*
**Education level n=827**				0.268
Up to primary	130 (30.4)	129 (32.3)	259 (31.3)	
Up to secondary	289 (67.7)	257 (64.3)	456 (66.0)	
Higher education	8 (1.9)	14 (3.5)	22 (2.7)	
**Single n=826**	276 (64.8)	299 (74.8)	575 (69.6)	0.002*
**Unemployed n=825**	235 (55.0)	213 (53.5)	448 (54.3)	0.662
**Household size n=802**				0.031*
0–2 individuals	214 (51.7)	230 (59.3)	444 (55.4)	
>2 individuals	200 (48.3)	158 (40.7)	358 (44.6)	
**Income categories ZAR n=730**				<0.001*
0	21 (5.5)	9 (2.6)	30 (4.1)	
1–1600	248 (64.8)	160 (46.1)	408 (55.9)	
1601–3200	71 (18.5)	103 (29.7)	174 (23.8)	
3201–6400	34 (8.9)	62 (17.9)	96 (13.2)	
6401–12 800	8 (2.1)	12 (3.5)	20 (2.7)	
≥12 801	1 (0.3)	1 (0.3)	2 (0.3)	
**Binge drinking among drinkers**	425 (97.0)	403 (97.3)	828 (97.8)	0.784
**Current smoker n=828**	123 (28.7)	90 (22.5)	213 (25.7)	0.04*
**Prison history n=837**	21 (4.9)	42 (10.3)	63 (7.5)	0.003*
**Miner (past or present) n=833**	17 (4.0)	7 (1.7)	24 (2.9)	0.053
**Health care worker**	8 (1.9)	7 (1.7)	15 (1.8)	0.889
**TB contact n=836**	54 (12.6)	51 (12.6)	105 (12.6)	0.999
**Previous TB n=830**	196 (46.0)	129 (31.9)	325 (39.2)	<0.001*
**Previous diabetes n=838**	19 (4.4)	20 (4.9)	39 (4.7)	0.717
**HIV-1 status**				<0.001*
Uninfected	160 (36.5)	121 (29.2)	281 (33.0)	
Infected	242 (55.3)	279 (67.4)	521 (61.2)	
Unknown	36 (8.2)	14 (3.4)	50 (5.9)	
**ART (among HIV-1-infected) n=521**	166 (68.6)	89 (31.9)	255 (48.9)	<0.001
**Previous gestational diabetes (among females) n=399**	4 (1.6)	8 (4.6)	12 (3.0)	0.105
**Hypertension**	154 (35.2)	75 (18.1)	229 (26.9)	<0.001*
**BMI kg·m^−2^ n=810**				<0.001*
<18.5 (underweight)	27 (6.6)	41 (10.3)	68 (8.4)	
18.5–24.9 (normal)	224 (54.4)	277 (69.6)	501 (61.9)	
25–29.9 (overweight)	69 (16.8)	59 (14.8)	128 (15.8)	
≥30 (obese)	92 (22.3)	21 (5.3)	113 (14.0)	
**Wide waist circumference^¶^ n=765**	144 (28.9)	53 (14.3)	167 (21.8)	<0.001*

Data are presented as n, n (%) or median (interquartile range), unless otherwise stated. ART: antiretroviral therapy; BMI: body mass index. ^#^: total n=852, unless otherwise stated in column 1; ^¶^: ≥94 cm males, ≥88 cm females. *: p<0.05.

### Prevalence of diabetes and IGR

The overall prevalence of diabetes (using any of the three diagnostic criteria) was 11.3% (95% CI 9.3–13.6%); 12.6% among TB cases (95% CI 9.7–16.1%) and 10.1% (95% CI 7.6–13.2%) in non-TB participants (p=0.246). Among diabetes participants, 59.4% (57 out of 96) were not previously diagnosed with diabetes (61.5% in TB cases and 56.8% in non-TB) and all previously diagnosed diabetes patients were on diabetes treatment. Despite treatment, participants with a prior diabetes diagnosis had higher median (IQR) FPG (7.45 (5.2–11.3) *versus* 5.45 (5.1–7.1) mmol·L^−1^; p<0.001) and HbA1c (9.7% (7.0–11.4%) *versus* 6.5% (6.4–6.9%); p<0.001) levels compared with newly diagnosed diabetes patients.

The overall statistically significant difference in glycaemic status between participants with and without TB was driven by the prevalence of IGR, which was significantly higher in TB cases than non-TB (65.2% *versus* 50.0%; p<0.001). The prevalence of diabetes and IGR varied by diagnostic test (table 2). The majority of diabetes diagnoses were made based on the FPG and HbA1c tests, while IGR was largely driven by a positive HbA1c test.

**TABLE 2:** Prevalence of diabetes and impaired glucose regulation (IGR), including previously diagnosed diabetes, by diagnostic test

	**Overall**	**Non-TB**	**TB**	**p-value (Chi-squared)**
**Diabetes**				
Any test	11.3 (9.3–13.6)	10.1 (7.6–13.2)	12.6 (9.7–16.1)	0.246
FPG	4.1 (3.0–5.7)	3.9 (2.4–6.2)	4.4 (2.8–6.9)	0.752
OGTT	3.3 (2.3–4.8)	3.5 (2.1–5.8)	3.1 (1.7–5.3)	0.704
HbA1c	8.2 (6.5–10.2)	6.2 (4.3–8.9)	10.2 (7.7–13.6)	0.032*
**IGR (excluding diabetes)**				
Any test	57.3 (53.7–60.8)	50.0 (45.1–54.9)	65.2 (60.0–70.0)	<0.001*
FPG	10.6 (8.6–13.1)	12.5 (9.5–16.1)	8.6 (6.1–12.2)	0.089
OGTT	10.6 (8.6–13.0)	4.9 (3.1–7.5)	16.9 (13.3–21.2)	<0.001*
HbA1c	39.5 (36.1–43.0)	34.1 (29.6–38.9)	45.4 (40.3–50.6)	0.002*

Data are presented as % (95% CI), unless otherwise stated. TB: tuberculosis; FPG: fasting plasma glucose; OGTT: oral glucose tolerance test; HbA1c: glycated haemoglobin. *: p<0.05.

The overall prevalence of diabetes was lower in HIV-1-infected patients compared with those uninfected or those with HIV-1 status unknown (8.9% *versus* 16.0% or 10.0%, respectively) (table 3). While there was no association between TB and diabetes overall, when stratified by HIV-1 status, the prevalence of diabetes was found to be higher in HIV-1-infected TB cases *versus* HIV-1-infected participants without TB (11.1% *versus* 6.2%; p=0.049). This difference was not observed in HIV-1-uninfected participants (table 3).

**TABLE 3:** Diabetes prevalence in tuberculosis (TB) and non-TB participants, stratified by HIV-1 status

	**Overall**	**Non-TB**	**TB**	**p-value**
**Subjects**	852	438	414	
**HIV-1-uninfected n=281**	16.0 (12.2–20.8) (n=45/281)	16.9 (11.8–23.6) (n=27/160)	14.9 (9.5–22.5) (n=18/121)	0.651
**HIV-1-infected n=521**	8.9 (6.7–11.6) (n=46/521)	6.2 (3.8–10.1) (n=15/242)	11.1 (7.9–15.4) (n=31/279)	0.049*
**HIV-1 status unknown n=50**	10.0 (4.1–22.4) (n=5/50)	5.6 (1.3–20.8) (n=2/36)	21.4 (6.0–54.0) (n=3/14)	0.093

Data are presented as n or % (95% CI), unless otherwise stated. *: p<0.05.

### TB/diabetes association and population-attributable risk

On univariable analysis, age, sex, marital status, household size, income, employment status, previous TB, HIV-1, smoking, hypertension, waist circumference, a history of time in prison, education, BMI and a history of being a miner were all associated with TB at the 10% level of significance. These variables were used in turn to build the multivariable model. After adjusting for confounding variables, diabetes was associated with a 2.4-fold higher odds of TB (95% CI 1.1–5.2), with this association remaining significant in HIV-1-infected but not HIV-1-uninfected individuals (table 4). Further analysis by diabetes diagnostic test revealed that this significant association was only noted using the HbA1c test. IGR was also associated with a 2.3-fold higher odds of TB (95% CI 1.6–3.3), with the strongest association shown when the OGTT was used as a diagnostic test compared with other tests (table 4). Unlike the diabetes analysis, the association between TB and IGR (using any test) was significant in both HIV-1-infected and -uninfected individuals, albeit driven by different tests: a significant association using the OGTT in HIV-1-infected individuals and the HbA1c test in HIV-1-uninfected individuals. Based on a 12% prevalence of diabetes in the general population and the adjusted OR of 2.4, the population-attributable risk of TB due to diabetes is 14%.

**TABLE 4:** Univariable and multivariable analysis of the association between tuberculosis and diabetes/impaired glucose regulation (IGR)

	**Overall**				**HIV-1-infected**				**HIV-1-uninfected**			
	**Crude OR (95% CI)**	**p-value**	**Adjusted OR^#^ (95% CI)**	**p-value**	**Crude OR (95% CI)**	**p-value**	**Adjusted OR^#^ (95% CI)**	**p-value**	**Crude OR (95% CI)**	**p-value**	**Adjusted OR^#^ (95% CI)**	**p-value**
**Diabetes**												
Overall	1.3 (0.8–2.0)	0.247	2.4 (1.3–4.3)	0.005*	2.0 (1.0–3.8)	0.033	2.4 (1.1–5.2)	0.030*	1.0 (0.5–1.9)	0.995	2.4 (0.9–6.7)	0.081
HbA1c	1.7 (1.0–2.9)	0.034	2.4 (1.2–4.6)	0.012*	2.2 (1.1–4.6)	0.032	2.4 (1.0–5.9)	0.05*	1.5 (0.7–3.2)	0.268	2.2 (0.7–6.4)	0.160
FPG	1.1 (0.6–2.2)	0.725	2.3 (0.9–5.5)	0.068	1.8 (0.5–6.0)	0.336	2.9 (0.7–12.2)	0.148	1.1 (0.5–2.6)	0.800	1.9 (0.6–6.4)	0.295
OGTT	0.9 (0.4–1.9)	0.704	1.2 (0.5–3.3)	0.690	1.8 (0.5–6.2)	0.322	2.5 (0.6–10.3)	0.218	0.5 (0.2–1.7)	0.285	0.5 (0.1–3.1)	0.491
**Previously diagnosed diabetes only**	1.1 (0.6–2.1)	0.717	3.7 (1.5–9.1)	0.004*	2.1 (0.5–8.3)	0.283	6.3 (1.3–30.8)	0.022*	1.2 (0.6–2.6)	0.652	3.1 (0.9–10.1)	0.066
**IGR (excludes diabetes cases)**												
Overall	1.9 (1.4–2.5)	<0.001	2.3 (1.6–3.3)	<0.001*	2.4 (1.7–3.4)	<0.001	2.4 (1.5–3.8)	<0.001*	1.2 (0.7–2.0)	0.467	2.3 (1.1–4.7)	0.024*
HbA1c	1.4 (1.0–1.8)	0.026	1.6 (1.1–2.3)	0.009*	1.6 (1.1–2.3)	0.012	1.5 (1.0–2.3)	0.084	1.1 (0.7–1.8)	0.569	2.2 (1.1–4.1)	0.017*
FPG	0.8 (0.5–1.1)	0.182	0.9 (0.5–1.5)	0.695	1.0 (0.6–1.7)	0.867	1.2 (0.6–2.2)	0.588	0.4 (0.2–0.8)	0.018	0.4 (0.1–1.2)	0.116
OGTT	3.0 (1.9–4.8)	<0.001	3.9 (2.1–7.0)	<0.001*	5.0 (2.6–9.5)	<0.001	5.4 (2.4–12.0)	<0.001*	1.4 (0.6–2.9)	0.432	2.8 (1.0–8.0)	0.058

HbA1c: glycated haemoglobin; FPG: fasting plasma glucose; OGTT: oral glucose tolerance test. ^#^: adjusted for sex, age, household size, income, hypertension (baseline), previous miner, previous prisoner, marital status, work status and HIV-1 status. *: p<0.05.

## Discussion

The prevalence of diabetes, using any three criteria, was 13% in TB cases in Cape Town, largely contributed to by HbA1c. There was also an alarmingly high prevalence of IGR, with 65% all TB cases having IGR. There is good evidence that TB is associated with transient hyperglycaemia, best measured using FPG and 2-h OGTT (markers of short-term glucose status), unlike HbA1c, which is a marker of glycaemic control over 2–3 months. The finding of the prevalence of diabetes being highest using HbA1c is therefore surprising. One possible explanation is the choice of cut-point. The American Diabetes Association/World Health Organization cut-point is 6.5% [30, 31], but a recent study investigating the diagnostic accuracy of HbA1c against the OGTT in a population survey of black South Africans suggested that the optimal cut-point for detection of diabetes was 6.0% [35]. However, using this lower cut-point would have resulted in a higher, not lower, prevalence. Similarly, anaemia, common in TB patients, may have resulted in lower HbA1c in TB cases, unless there was iron deficiency with or without anaemia which may result in an increase in HbA1c compared with non-TB participants, without a concomitant rise in glucose indices [36]. It may be that use of HbA1c in the context of TB and/or HIV-1 is flawed, or that the cut-point should be revised. Further research is required to better understand factors, such as altered red cell survival, that may influence HbA1c in TB/HIV-1 patients.

We noted a significant association between diabetes and TB, in agreement with the majority of the published literature from other settings, with a 2.4- and 2.3-fold higher odds of TB in diabetes and IGR patients, respectively, and 14% of TB cases attributed to diabetes. Only two of these studies were conducted in a setting with a high HIV-1 burden [21, 37] with ORs ranging from 2.2 to 10.7. The coexistence of a high prevalence of HIV-1, TB and diabetes has significant implications for optimal control of each condition, highlighting the importance of targeting TB control interventions, such as intensified TB screening for diabetes and diabetes/HIV-1 patients, to achieve TB elimination goals.

There was also a significant TB/IGR association (adjusted OR 2.3), suggesting that a finding of IGR should also prompt TB screening. While the high prevalence of IGR would support the theory of these cases being transient hyperglycaemia, the finding of the high prevalence strongly driven by HbA1c is a conundrum as it would suggest a more sustained state of hyperglycaemia. Negative outcomes have been associated with TB/diabetes [3]. HIV-1-uninfected TB/diabetes patients are at higher risk of death than TB/HIV patients [38, 39], but there is a paucity of research on the association between IGR and TB outcomes to inform the need for interventions to control blood glucose in IGR TB patients.

The association between diabetes and TB also varied depending on the diagnostic test used. We found that the association was largely driven by the HbA1c test, as did Boillat-Blanco *et al.* [37] in Tanzania. Using either FPG or OGTT alone as a diabetes diagnostic test did not reveal a statistically significant association. Other studies from Tanzania and Guinea-Bissau also found that IGR/diabetes diagnoses varied when using random blood glucose, FPG and OGTT [19, 21]. Further research is required to adequately define the most appropriate test to identify and monitor diabetes in TB patients, including in HIV-1-co-infected individuals.

On stratification by HIV-1 serostatus, the association between TB and diabetes remained statistically significant only in participants with HIV-1 infection. The reported effect of HIV-1 co-infection on the association between TB and diabetes has been variable. A study using random blood glucose reported reduced odds of diabetes in HIV-1-infected patients [20]. Another study using FPG and OGTT reported similar findings of a stronger TB/diabetes association in HIV-1-uninfected patients [21]. However, a recent study that used all three diabetes diagnostic tests showed a slightly stronger TB/diabetes association among HIV-1-infected individuals, as measured by FPG and OGTT, but not by HbA1c [37]. In our study, the significant TB/diabetes association in HIV-1-infected participants was noted when diabetes was defined using any of the three tests, largely driven by HbA1c. There are limited data on the effect on HIV-1 infection on HbA1c. One study that compared HbA1c values in HIV-1-infected and -uninfected women found slightly lower HbA1c values in HIV-1-infected women after adjustment for fasting glucose values [40]. However, this association was nullified on multivariable analysis, with the differences accounted for by higher red cell mean corpuscular volume in HIV-1-infected individuals. Another study also suggested that HbA1c may underestimate glycaemia in HIV-infected individuals, particularly those with higher mean corpuscular volume, nucleoside reverse transcriptase inhibitor use and lower CD4 count [41].

The association between TB and diabetes was stronger in participants with a prior diabetes diagnosis, with a 3.9-fold higher odds of TB. These participants had a higher median FPG and HbA1c, and thus the stronger association may reflect the greater degree of hyperglycaemia in this group. This is in line with published studies that have shown that poorly controlled diabetes is more strongly associated with TB [42]. Of note, HIV-1-infected patients with a previous diabetes diagnosis were at an even greater risk of TB with the adjusted OR increasing from 2.4 to 6.3. In this and other studies [13, 20], a high percentage of diabetes among TB patients was previously undiagnosed. Data from chronic disease audits across more than 100 primary care facilities in the Western Cape Province of South Africa, within which Cape Town is located, have reported that <15% of diabetes patients attending clinics have HbA1c levels <7% (Western Cape Dept of Health, Cape Town, South Africa; unpublished data). Our results therefore strongly support the need to prioritise improved diabetes management as a TB control interventions.

### Strengths and limitations

A significant strength of this study was the performance of three different tests for diabetes using venous blood samples with all tests performed in an accredited national laboratory. In addition, multivariable analysis of the association between TB and diabetes/IGR adjusted for the most prevalent and significant known confounders, including a previous history of time in prison, mine work and household income, as well as HIV-1 infection.

Our study had a number of limitations. Glycaemia was measured at baseline and it is possible that the hyperglycaemia was transient. We attempted to limit the effect of transient hyperglycaemia on the reported TB/diabetes association by selecting non-TB participants from patients with respiratory symptoms that later resolved. Studies have shown an association between hyperglycaemia at TB diagnosis (even when transient) and TB treatment failure and death [37], highlighting the clinical importance of intervening in patients with baseline hyperglycaemia to improve TB outcomes. Small numbers after stratification by HIV-1 status meant we were unable to investigate the effect of ART or CD4 count. In addition, given that the association between TB and diabetes was significant overall, and that the study was powered to detect this association, the lack of statistical significance noted in HIV-1-uninfected participants may be due to a reduced statistical power after stratification; this study is not able to exclude the possibility of an association in HIV-uninfected individuals. Finally, given the association between diabetes and poorer TB outcomes, the exclusion of critically ill patients due to their inability to give consent may have introduced selection bias with possible exclusion of TB patients with diabetes. Similarly, while the multidrug-resistant (MDR)-TB prevalence found in this study is similar to the national prevalence (2.8%) [43], given the poor outcomes associated with MDR-TB, the exclusion of critically ill patients may also have resulted in a greater proportion of excluded patients with MDR-TB.

### Conclusion

Diabetes is a significant contributor to the TB burden in this high TB/HIV-1/diabetes burden setting. Our study utilised international guideline-approved diabetes tests and found a high prevalence of diabetes in TB cases, and a significant association between TB and diabetes, even in the context of HIV-1 co-infection, with greater odds of TB in participants who were previously diagnosed with diabetes. These findings point to the importance of screening for diabetes in those with TB and those with HIV-1, who are already at high risk for developing TB. A surprisingly high prevalence of IGR was also found in this study, emphasising the need for further investigation into the TB outcomes and management of this group. The interpretation of our findings is complex because of the variations in diabetes prevalence and TB/diabetes association by different diagnostic tests. Existing glycaemia measures can be affected by HIV-1, ART, anaemia, acute infection, red cell survival and iron status. This highlights the need for more accurate markers of glycaemia that are independent of these factors in order to inform policy on how best to screen for diabetes at the time of TB diagnosis in (often low-resource and primary care) settings with a high burden of HIV-1 and TB.
